# Heart failure and Alzheimer′s disease

**DOI:** 10.1111/joim.12287

**Published:** 2014-08-01

**Authors:** P Cermakova, M Eriksdotter, L H Lund, B Winblad, P Religa, D Religa

**Affiliations:** 1 Division for Neurogeriatrics, Department of Neurobiology, Care Sciences and Society, Center for Alzheimer Research, Karolinska InstitutetHuddinge, Sweden; 2 International Clinical Research Center and St. Anne's University HospitalBrno, Czech Republic; 3 Department of Geriatric Medicine, Karolinska University HospitalStockholm, Sweden; 4 Division of Clinical Geriatrics, Department of Neurobiology, Care Sciences and Society, Center for Alzheimer Research, Karolinska InstitutetStockholm, Sweden; 5 Department of Cardiology, Karolinska University HospitalStockholm, Sweden; 6 Unit of Cardiology, Department of Medicine, Karolinska InstitutetStockholm, Sweden; 7 Department of Medicine, Center for Molecular Medicine, Karolinska InstitutetStockholm, Sweden

**Keywords:** Alzheimer′s disease, dementia, heart failure, neurocardiology, neurovascular unit

## Abstract

It has recently been proposed that heart failure is a risk factor for Alzheimer′s disease. Decreased cerebral blood flow and neurohormonal activation due to heart failure may contribute to the dysfunction of the neurovascular unit and cause an energy crisis in neurons. This leads to the impaired clearance of amyloid beta and hyperphosphorylation of tau protein, resulting in the formation of amyloid beta plaques and neurofibrillary tangles. In this article, we will summarize the current understanding of the relationship between heart failure and Alzheimer′s disease based on epidemiological studies, brain imaging research, pathological findings and the use of animal models. The importance of atherosclerosis, myocardial infarction, atrial fibrillation, blood pressure and valve disease as well as the effect of relevant medications will be discussed.

## Introduction

Dementia and heart failure (HF) both represent growing social, healthcare and economic problems. It is estimated that there were more than 35 million persons worldwide with dementia in 2010, and this number is expected to double every 20 years [1], largely due to an ageing population but also to an increasing prevalence of risk factors for dementia. The annual worldwide costs of dementia were 604 billion dollars in 2010 [2]. The most common form of dementia is Alzheimer′s disease (AD), and the major risk factor for its development is increasing age [3]. Other known risk factors include family history, hypertension and hypotension, high cholesterol levels, low levels of physical activity and of education, obesity, and the presence of epsilon 4 alle of the apolipoprotein E gene (*APOE4*) [4–6]. A recently proposed risk factor for AD is HF [7].

Chronic HF is a progressive condition that may be defined as inadequate cardiac output to meet metabolic demands. The most common causes of HF in developed countries include ischaemic heart disease with myocardial infarction, hypertension, cardiomyopathy and degenerative valve disorders.

The prevalence of HF is about 2% [8], increasing sharply with age, with up to 10% of individuals over 65 years [8] and 20% over 75 years affected [9]. It has been shown that HF is more ‘malignant’ cancer overall [9]. Hospitalization for HF accounts for 1–2% of all healthcare expenditure in Europe [10], and HF is the most common cause of hospitalization in patients over 65 years of age [11].

Alzheimer′s disease and HF often occur together and thus increase the cost of care and health resource utilization [12]; this highlights the need to investigate the relationship between these two conditions. Impaired cognition in HF patients leads to significantly more frequent hospital readmissions [13] and increases mortality rates [14].

The relationship between HF and AD remains largely unclear. In this review, we aim to explain how HF contributes to the development of AD, focusing mainly on reduced cerebral blood flow (CBF) [15] and dysfunction of the neurovascular unit [16]. However, multiple cardiovascular conditions often coexist, suggesting that several mechanisms underlying cardiovascular dysfunction may contribute to cognitive decline. HF in the elderly is often underdiagnosed, because the symptoms of HF are mimicked or masked by comorbidities in this population [17]. Causes of HF and common comorbidities will be discussed with emphasis on their contribution to dementia and specifically AD.

Early identification and correct medical treatment of cardiovascular conditions can reduce the prevalence of AD [18]. Indeed prevention of AD may be more effective than current pharmacological treatment [19,20]. It has been estimated that delaying the onset of AD by just 1 year would lead to 9 million fewer cases by 2050 [21]. During the past 3 years, the results of at least five studies have been published suggesting that the incidence of dementia and AD may have decreased over the last two decades [22–26]. The mortality improvements are generally attributed to better awareness of cardiovascular disease risk factors. In addition, successful management of hypertension and increase in the use of statins and antithrombotic drugs may play an important role.

## Cognitive disorders: dementia and delirium

Cognition is a group of mental processes which include memory, attention, learning, decision making, problem solving, language processing and executive functions. Dementia is a progressive cognitive disorder which usually primarily affects memory. In addition, aphasia, apraxia, agnosia and disturbances of executive functioning are common symptoms. Delirium, on the other hand, is characterized by an abrupt impairment of cognition and a fluctuating course. It is very common amongst elderly patients undergoing a surgery [27]. Precipitating insults include infection, intoxication, high volume load, electrolyte imbalance, fever or administration of anticholinergic drugs [28,29]. Delirium has been generally viewed as an acute state, but may in fact persist for several months [30]. Delirium and dementia often coexist [31]; indeed, delirium may be both a prognostic factor for dementia and worsen cognitive impairment in pre-existing dementia [32].

## HF with preserved ejection fraction

Heart failure was originally a clinical diagnosis that relied on classical symptoms and signs of neurohormonal activation, in particular fluid retention, as summarized in the Framingham criteria. The availability of and over-reliance on echocardiography led to a focus on reduced ejection fraction, generally considered to be <40–50%, as a tool and sometimes even a criterion for diagnosis. However, it has become increasingly clear that HF also occurs with preserved ejection fraction and is equally common and perhaps equally serious [33]. Because HF with preserved ejection fraction is associated with diastolic dysfunction, that is, impaired active relaxation and passive filling during diastole, it is often termed diastolic HF [34], but impaired systolic contractility occurs, too [35]. HF with preserved ejection fraction is associated with older age, female gender, hypertension and atrial fibrillation. There is continuing debate as to whether HF with preserved ejection fraction is a distinct syndrome or a consequence of ageing and the associated subtle renal insufficiency, vascular changes and anaemia [36]. However, given the multifactorial nature of this syndrome and its close correlation with age and other risk factors for dementia, HF with preserved ejection fraction is likely to be highly relevant for the development of dementia even though causality may be difficult to establish.

## HF and cognition

Since 1977 when the term ‘cardiogenic dementia’ was first introduced [37], it has been confirmed that HF contributes to cognitive decline [38] and the grade of cognitive impairment correlates with the severity of HF [39]. Even though heart disease and AD share similar genetic backgrounds and risk factors such as *ApoE* polymorphisms, it is becoming increasingly clear that there is also an association through their dependency on an adequate blood supply. Insufficient blood circulation may contribute to changes in all organs and lead to the multiple organ dysfunction syndrome. Decreased cardiac output due to HF is associated with abnormal brain ageing and cognitive impairment [40]. Data from the Framingham Heart Study confirmed that reduced cardiac index and left ventricular ejection fraction are associated with impaired cognition [41]. Lower values of cardiac index were even found to be related to smaller brain volumes [42]. Findings of other studies demonstrated that left ventricular ejection fraction is linked to cognitive decline in patients with HF [43]. A low left ventricular ejection fraction was related to memory [44], reasoning and sequencing impairment [45].

Data from a recent study demonstrate some degree of cognitive decline in almost 47% of patients hospitalized for HF [46]. HF increases the risk of delirium [28] and, on the other hand, delirium is associated with a more advanced stage of HF [47]. Few studies have investigated the prevalence of dementia or its subtypes in subjects with HF. Recently, in a Swedish population-based longitudinal study, it was found that 40% of patients with HF also had dementia. By contrast, dementia was present in 30% of individuals without HF [48]. There is growing evidence that HF is a risk factor for both vascular dementia and AD [7,49], but the prevalence of AD in HF patients is not known.

## HF and structural brain changes

Growing evidence from neuroimaging studies suggests an association between HF and structural brain abnormalities, which further supports a relationship between dysfunction of both the heart and brain. Total and regional brain atrophy or demyelination are common in patients with HF [50]; indeed, Kumar and colleagues found reduced axonal integrity of several brain circuits that are involved in cognition in these patients [51]. Serber *et al*. [52] reported abnormalities of the frontal cortex which were correlated with reduced cognitive functioning.

In a study comparing HF patients with both healthy control subjects and patients diagnosed with heart disease other than HF, it was found that HF was related to more white matter hyperintensities, lacunar infarcts and medial temporal lobe atrophy. Medial temporal atrophy is an early feature of AD and is associated with lower cognitive function and an increased risk of progression to dementia [53]. However, these pathological findings were more likely to be found even in patients with heart disease other than HF compared with healthy individuals, thus cannot be considered as a specific consequence of HF [50].

Considerable data suggest that heart disease may precipitate neurodegenerative changes seen as white matter hyperintensities [54], which have been associated with cognitive functions [55]. However, the clinical significance of these changes is not clear, as they are extremely common in the elderly population and in many studies were not related to cognitive performance [56,57].

## CBF in AD

It has been demonstrated extensively that vascular changes and reduced blood supply of the brain are involved in the pathogenesis of AD [58]. There is a large body of evidence demonstrating that AD is characterized by reduction in both total and regional CBF with resulting brain hypoperfusion. Total CBF is about 20% lower in patients with AD compared with individuals without dementia [15]. Lower CBF has been reported and confirmed in many studies using single-photon emission computed tomography (SPECT), positron emission tomography, spin-labelling magnetic resonance imaging (MRI) or transcranial Doppler measurements [59–61]. It seems likely that reduced CBF can cause neuronal dysfunction or death [62].

Results of the Rotterdam Study, a prospective population-based cohort with 1730 participants, suggested that cerebral hypoperfusion precedes and possibly contributes to the onset of clinical dementia [63]. This is supported by the findings of a delay in CBF response in patients with mild cognitive impairment (MCI) and an even longer delay in patients with AD in a study using functional MRI and blood oxygenation level dependent contrast [64]. Because MCI may be considered the earliest clinical feature of AD, this evidence suggests that CBF reductions are present in early stages of AD pathogenesis. This is consistent with results from a longitudinal study using SPECT to investigate CBF in MCI patients with a high predictive value for conversion to AD. Significant reductions in the parietal lobule, angular gyrus and precuneus were found [65], which implies that reduced CBF precedes neurodegeneration.

Furthermore, decreased CBF may negatively affect the synthesis of proteins required for memory and learning and may eventually contribute to neuritic injury and neuronal death [66]. It has been suggested that brain hypoperfusion may be caused by the continuous loss of cholinergic innervation of intracerebral blood vessels [67].

## CBF and HF

In the presence of HF as well as older age, the mechanisms that regulate changes in CBF become compromised [68]. Reduced CBF was observed in patients with HF, and a correlation with an increasing prevalence (of up to 25%) of cognitive dysfunction was found [69]. Improvement in heart function following transplantation or resynchronization increased levels of CBF and cognitive function [45,70,71].

There is growing evidence that reduced CBF due to HF has a role in both pathological hallmarks of AD: amyloid beta (Aβ) deposition and tau protein aggregation [72].

## Aß and tau protein in HF

Reduced CBF compromises the oxygenation of neurons. In HF, neurons are chronically exposed to an insufficient blood supply. Therefore, HF does not result in sudden neuronal death; instead, neurons undergo a metabolic energy crisis. The lack of energy results in acidosis and oxidative stress followed by a cascade of pathological consequences such as dysfunction of enzymes and protein synthesis. It has been shown that brain acidosis is associated with aggregation of both the altered tau and Aβ [73]. Furthermore, an acidic environment stimulates autoactivation of the lysosomal enzyme asparaginyl endopeptidase, which cleaves inhibitor 2 of protein phosphatase 2A [74]. Phosphatase 2A accounts for most of the tau protein phosphatase activity; therefore, its disinhibition results in hyperphosphorylation of tau [75]. Abnormal hyperphosphorylated tau protein loses the ability to bind to tubulin to promote its assembly into microtubules. Instead, it binds to normal tau protein, which leads to formation of tau oligomers and their aggregation into neurofibrillary tangles [76,77].

Lack of energy has also been shown to upregulate beta secretase 1, a protease responsible for cleavage of the amyloid precursor protein [78]. This results in accumulation of Aβ protein and formation of amyloid plaques. However, the increased processing of amyloid precursor protein is not the only mechanism involved in AD pathology. It has been suggested that a more important mechanism in HF is impaired Aβ clearance across the blood–brain barrier (BBB) [66].

## BBB and impaired Aßclearance

The BBB consists of endothelial cells connected by tight junctions and a thick basement membrane which is supported by astrocytic end feet. The BBB represents an important part of a functional cellular structure known as the neurovascular unit, which also includes pericytes and microglia [79]. Dynamic communication between the cells of the neurovascular unit is required to enable efficient clearing of Aβ to prevent it from accumulating in plaques [80]. Impaired cerebral blood supply can lead to failure in maintaining adequate oxygenation of the cells [58]. Hypoxia results in regressive changes and can cause the breakdown of the BBB which impairs the clearance of Aβ.

**Figure 1:**
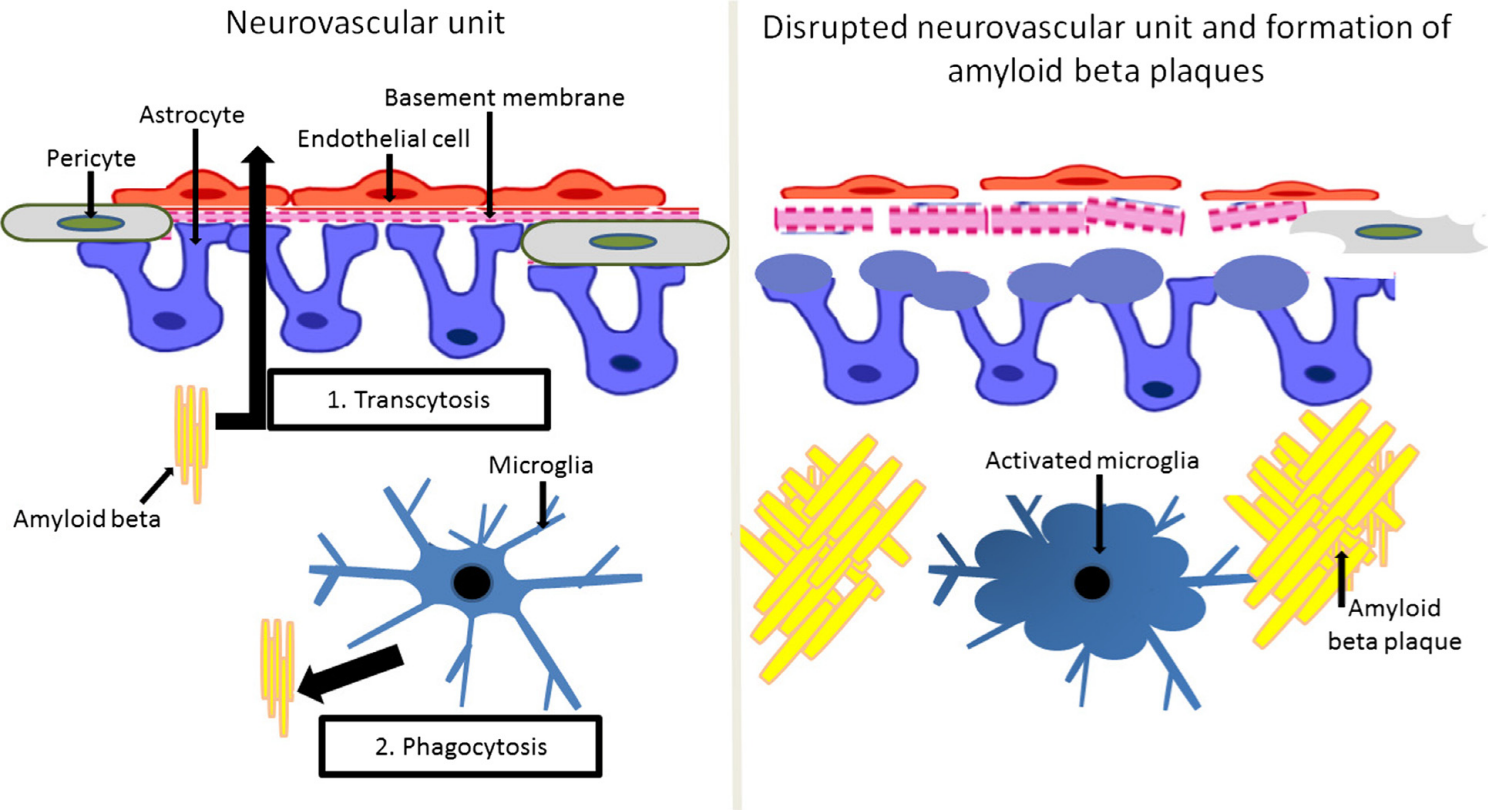
The neurovascular unit, amyloid beta clearance and formation of amyloid beta plaques. The blood–brain barrier is composed of endothelial cells supported by a basement membrane and astrocytic feet. The blood–brain barrier represents an important part of a functional structure known as the neurovascular unit, which also includes pericytes and microglia. Amyloid beta is cleared by transcytosis through the cells of the blood–brain barrier and by phagocytosis by microglia. Disruption of the neurovascular unit is characterized by dysfunctional endothelial cells with abnormal intercellular connections, thickening and rupture of the basement membrane, swelling of astrocytic end feet and activated pericytes and microglial cells. The breakdown of the blood–brain barrier inhibits amyloid beta clearance via transcytosis. Activated microglia are not able to phagocytose its excess which results in accumulation of amyloid beta plaques.

There are several ways in which Aβ is cleared across the BBB from brain parenchyma into the blood [66]; two of these are discussed here (Fig.1). The first mechanism involves transcytosis through the cells of the BBB, and the second represents phagocytosis by microglia [81]. Microglia play a major role in scavenging Aβ and their accumulation and activation is a typical feature of AD pathology [82]. Thus, it is beneficial to clear Aβ in the early stages of the disease [83]. However, during progression of the disease, activated microglia are further involved in inflammatory processes [84] and lose their ability to phagocytose Aβ [85].

The disruption of the neurovascular unit is characterized by many pathological events, as shown in Fig.1. Endothelial dysfunction seems to play a crucial role, as degeneration of endothelial cells is part of the disease process of AD [86]. Activation of endothelial cells and abnormal communication between these cells and pericytes is followed by the disruption of the basement membrane and swelling of the astrocytic end feet. Dysfunction of the BBB leads to infiltration of inflammatory cells, which increases oxidative stress [87]. Pericytes seem to contribute to destructive inflammatory responses [88]. Inflammation and oxidative stress compromise neuronal repair mechanisms [89,90] and further impair the phagocytic function of microglia.

Breakdown of the BBB results in the impaired clearance of Aβ via transcytosis, which leads to amyloid accumulation in the brain parenchyma and in and around capillaries; this is known as cerebral amyloid angiopathy (CAA). CAA, a major pathological insult to the neurovascular unit [91], is associated with cognitive impairment [92] and is present in more than 80% of patients with AD [93]. Okamoto *et al*. [93] found that cerebral hypoperfusion accelerates CAA, which is consistent with the hypothesis that a reduction in CBF precedes AD pathology.

**Figure 2:**
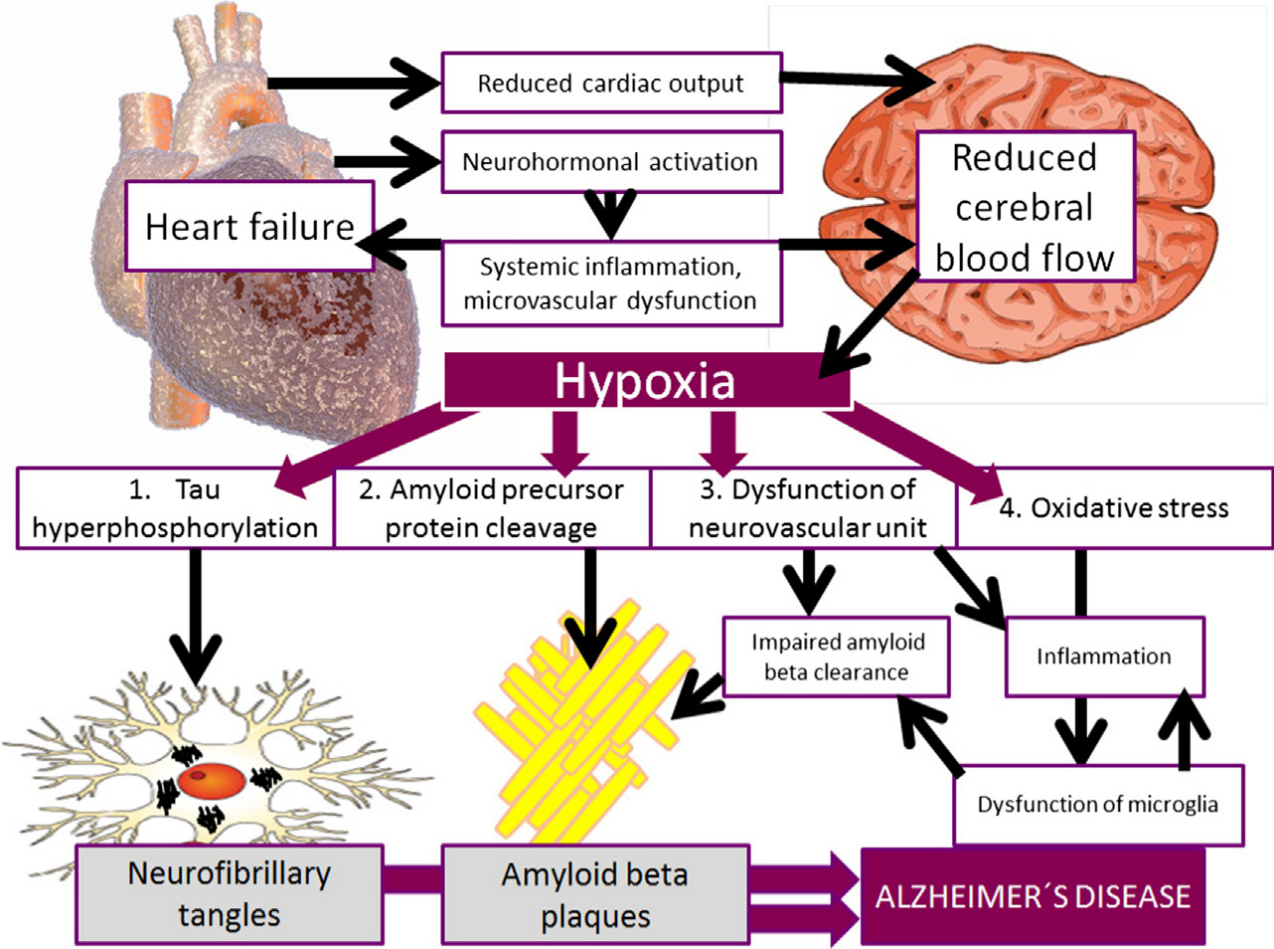
Model of the relationship between heart failure and Alzheimer′s disease. This model shows the possible direct and indirect pathways of the contribution of heart failure to the development of Alzheimer′s disease. Low cardiac output may directly lead to reduced cerebral blood flow. Neurohormonal activation, inflammation and microvascular dysfunction may indirectly contribute to impaired perfusion and therefore insufficient oxygenation of the brain. Hypoxia induces hyperphosphorylation of tau protein and expression of beta secretase which cleaves amyloid precursor protein. In addition, insufficient blood supply causes disruption of cells comprising the neurovascular unit and induces oxidative stress.

Heart failure gives rise to compensatory neurohormonal activation which is adaptive and restores cardiac output in the short term, but leads to a vicious circle of progressive remodelling and further neurohormonal activation which is maladaptive in the long term. Major components of the progressive pathophysiology of HF are systemic inflammation, oxidative stress and impaired endothelial function [94]. Despite a lack of sufficient evidence, it seems likely that the systemic inflammatory state in HF has an impact on the neurovascular unit and contributes to its dysfunction (Fig.2).

## Animal models

A suitable animal model of AD is one that can be used for the analysis of vascular morphology, blood flow and memory impairment [95,96]. In a critical review of mouse models that are used to demonstrate effects of cardiovascular diseases on cognition, Bink and colleagues concluded that the most promising model is based on hypoperfusion caused by bilateral common carotid artery stenosis [97]. This animal model represents a chronic hypoperfusion of the brain caused by placing microcoils around the carotid arteries [98]. The dissection revealed activated microglial cells and astrocytes, white and grey matter changes, hippocampal atrophy and micro-infarcts [99–101]. These findings support a link between brain hypoperfusion and the development of AD [102,103].

In a second mouse model, ligation of the aortic arch between the carotid arteries demonstrated chronic HF leading to a reduced ejection fraction. This model showed increased permeability of the BBB and decreased CBF [104]. Using both mouse models, Aβ deposits were observed [105,106] supporting the association between cardiac dysfunction and AD pathology.

In experimental studies on rats, acute cessation of blood flow induced expression of diffuse Aß peptide and amyloid precursor protein in the hippocampus, entorhinal cortex and neocortex [107]. This further supports the above-mentioned theory that cerebral hypoperfusion places the brain at risk of amyloid deposition. Neurohormonal maladaptive changes and systemic inflammation have not been discussed in these articles.

## Blood pressure

Arterial hypertension is the most common comorbidity of and a critical risk factor for HF as well as an important contributor to the development of AD, independent of HF [108,109]. As HF increases in severity, patients often experience low or fluctuating blood pressure [110] that can impair cognition both acutely and in the long term. Hypotension in HF is associated with poor outcome [111] and predicts cognitive impairment amongst patients with HF [112]. A decline in blood pressure is observed several years prior to the diagnosis of AD which can be attributed to impaired cerebral autoregulation and vasomotor reactivity [113]. Therefore, it has been speculated that low blood pressure accelerates neurodegeneration [114,115]. Fluctuating blood pressure may be a result of systolic pump failure, treatment or nonadherence to dietary recommendations or medication [110,116]. In addition, it has been related to poor performance in cognitive tests, structural brain changes and white matter hyperintensities [117,118].

However, hypotension is a marker of advanced stage HF [119], where renal function and response to diuretics deteriorate, uraemia and systemic inflammation increase, and numerous neurohormonal and metabolic pathways are progressively and abnormally activated. Furthermore, increasing doses of neurohormonal antagonists are beneficial even in patients with low blood pressure [120], suggesting that whereas hypotension may contribute to dementia, neurohormonal antagonists may retard the progression of HF. Typically, up-titration of drugs is initially associated with lower blood pressure and increasing symptoms of fatigue, dizziness and possibly impaired cognition, but the long-term effects are protective and may retard dementia. Thus, balancing the use of HF drugs with aspects of frailty in patients with dementia becomes difficult.

Hypertension often precedes HF and contributes to the progression of this disorder [121]. Furthermore, hypertension is a major risk factor for the development of left ventricular hypertrophy and myocardial infarction which may result in HF [110]. In addition, midlife hypertension in particular is a risk factor for AD [4,122]. Correct management of blood pressure may therefore affect the emergence of AD in HF patients in a complex manner. However, the findings from studies of the relationship between hypertension and dementia in older age are not consistent [123]. Results from clinical trials that examined the preventive effect of antihypertensive therapy on dementia were not conclusive: whilst some studies proved a protective effect of some antihypertensive drugs on the prevention of dementia [123], this was not confirmed in other trials [124,125].

Furthermore, hypertension may produce additive vascular pathology through disruptions of both the BBB and CBF homoeostasis. The effect of hypertension can be related to increased vascular stiffness and acceleration of atherosclerosis [108,126].

## Atherosclerosis

Atherosclerosis is an important mechanism underlying conditions resulting in HF, such as coronary heart disease. Even in the absence of preceding myocardial infarction or macrovascular coronary disease, atherosclerosis, inflammation, endothelial dysfunction and microvascular remodelling are thought to be critical features of the pathophysiology of HF, in particular HF with preserved ejection fraction [127]. Furthermore, atherosclerosis is associated with a risk of developing dementia in the elderly [128]. Although atherosclerosis used to be attributed particularly to vascular dementia, it is now becoming increasingly evident that it contributes to the pathogenesis of AD [129].

However, it is not completely clear whether the observed effect of atherosclerosis on AD is due to direct vascular changes or whether there is an effect of common risk factors and underlying diseases. This relationship may be explained by the presence of ischaemia caused by disturbances in CBF due to progressive vascular narrowing [130]. However, atherosclerosis is a complex disease that involves several biochemical, immunological and inflammatory mechanisms that lead to damage of the vascular wall, and accumulation of cholesterol crystals and inflammatory cells [131]. This leads to progressive compensatory and adaptive vascular morphological changes and disruption of the neurovascular unit [132]. Atherosclerosis is also related to the formation of small intracerebral aneurysms that can underlay micro-bleedings observed in the brain of patients with AD [133].

Furthermore, atherosclerosis and AD share common risk factors, such as age [134], *ApoE4* polymorphisms [135], homocysteine [136], smoking [137], obesity [138] and chronic inflammation [139]. In addition, both atherosclerosis and AD are strongly associated with several underlying conditions, such as hypertension [4], diabetes mellitus [140] and hypercholesterolaemia [4]. The latter may explain the association between AD and extensive peripheral atherosclerosis [141,142].

A number of post-mortem studies have been conducted to investigate intracerebral atherosclerotic changes in patients with AD, as shown in Table1. According to Yarchoan and colleagues, 77% of individuals with AD had apparent atherosclerosis in the circle of Willis, compared with only 47% of control subjects. The association between atherosclerosis of the circle of Willis and AD pathology was more significant for women than men [143].

**Table 1:** Association between intracranial atherosclerosis and Alzheimer′s disease pathology: overview of autopsy studies

Reference	Number of participants (patients with AD; control subjects)	Mean age at death ± SD, years (patients with AD; control subjects)	Results
Beach *et al*. 2007 [144]	215; 92	82.6 ± 8.2; 84.3 ± 6.8	Relation to both Aß plaques and neurofibrillary tangles
Dolan *et al*. 2010 [149]	200;/	87.6 ± 7.1;/	No relation to AD pathology
Honig *et al*. 2005 [146]	676; 226	78.8 ± 8.8; 82.9 ± 10.0	Relation to Aß plaques, but not to neurofibrillary tangles
Kosunen *et al*. 1995 [147]	32; 6	84.0 ± 9.0; 82.0 ± 16.0	No relation to AD pathology
Roher *et al*. 2003 [145]	32; 22	85.2; 85.5	Relation to both Aß plaques and neurofibrillary tangles
Luoto *et al*. 2009 [237]	466;/	70.8 ± 46.9;/	No relation to AD pathology
Roher *et al*. 2011 [129]	61; 36	85.1 ± 7.3; 84.9 ± 6.1	Relation to both Aß plaques and neurofibrillary tangles
Yarchoan *et al*. 2012 [143]	410; 59	77.1 ± 10.5; 69.6 ± 15.9	Relation to Aß plaques, neurofibrillary tangles and CAA, particularly in women
Zheng *et al*. 2013 [238]	81; 23	84.9 ± 7.1; 84.6 ± 5.9	No relation to AD pathology

SD, standard deviation; AD, Alzheimer's disease; Aβ, amyloid beta; CAA, cerebral amyloid angiopathy.

Beach and co-workers demonstrated that the grade of atherosclerosis of the circle of Willis was more severe in cases with AD and vascular dementia compared with nondemented individuals [144]. The severity of cerebrovascular atherosclerosis of the circle of Willis was associated with an increased number of Aβ plaques and neurofibrillary tangles in two studies [144,145]. Honig *et al*. [146] also established a strong link between cerebral atherosclerosis and the density of Aβ plaques, but did not find any association with neurofibrillary tangles.

However, this evidence is inconsistent with the findings of several other studies in which a relation between atherosclerosis and AD was not confirmed. In a post-mortem study, Kosunen *et al*. [147] found no relationship between atherosclerotic changes and AD pathology. Similarly, in an autopsy study by Zheng and colleagues, there was no significant correlation between atherosclerosis and Aβ plaques or neurofibrillary tangles. By contrast, the authors showed that atherosclerosis was associated with micro-infarcts, which have previously been correlated with the grade of cognitive decline [148].

In the Baltimore Longitudinal Study of Aging, a prospective longitudinal study in which a complete autopsy was conducted in 170 participants, it was confirmed that the presence of atherosclerosis in intracranial vessels increases the risk of dementia independently from cerebral infarction, but no association was found between the degree of atherosclerosis and AD pathology [149].

## Coronary artery disease and myocardial infarction

Several studies have confirmed that coronary artery disease is associated with cognitive impairment [150,151], reduced hippocampal volume [152] and dementia [153]. Recently, Graban and colleagues showed that coronary artery disease was more common in patients diagnosed with vascular dementia compared with controls [154]. The results from the Rotterdam study demonstrated that unrecognized myocardial infarction was associated with a higher risk of dementia, increased white matter lesions and brain infarctions in men [155].

The Bronx Aging Study provided evidence that women with a history of myocardial infarction had a fivefold increase in the risk of dementia [156]. However, in some studies, such as the Honolulu-Asia Aging Study and the Rochester Epidemiology Project, no such associations with later cognitive impairment [157] or dementia [158] were found. Therefore, further studies are needed to investigate the link between AD and prior myocardial infarction.

## Atrial fibrillation

Atrial fibrillation (AF) is the most common type of arrhythmia. Its prevalence in HF ranges between 13% and 40% [159,160] and is even higher in HF with preserved ejection fraction [161]. It has been suggested that AF can precipitate HF, but a causative relationship has not been established [162]. In a large number of studies, scores in cognitive tests were reduced and learning, memory, attention and executive functions were impaired in individuals with AF [163–165]. Furthermore, neuroimaging studies have demonstrated a reduced hippocampal volume in AF patients [166]. It has been found that AF increases the risk of dementia [167,168] and predicts its development in subjects with cognitive impairment [169,170]. Surprisingly, in the Rotterdam study, AF was related more strongly to AD than vascular dementia [171], whilst Bunch *et al*. [172] found that it was associated with all types of dementia disorders. However, in other studies, no correlation between AF and dementia or cognitive decline was found [173,174]. Studies of the role of AF in dementia disorders are shown in Table2.

**Table 2:** Atrial fibrillation as a risk factor for dementia disorders in a stroke-free population: overview of studies

		Results			
Reference	Study design	AF and dementia	AF and AD	AF and vascular dementia	Conclusion
Bunch *et al*. 2010 [172] Intermountain Heart Collaborative Study	Longitudinal study	–	OR = 1.06; *P *=* *0.59	OR = 1.73; *P *=* *0.001	AF is associated more strongly with vascular dementia than with AD
	Follow-up for 5 years				
Dublin *et al*. 2011 [167]	Longitudinal study	HR = 1.38; 95% CI 1.10–1.73	HR = 1.50; 95% CI 1.16–1.94	–	AF is associated more strongly with AD than with all-cause dementia
	Follow-up for 6.8 years				
Forti *et al*. 2006 [239]	Longitudinal study	HR = 1.10; 95% CI 0.40–3.03	–	–	AF is not associated with dementia in a cognitively normal population
	Follow-up for 4 years				
Marengoni *et al*. 2011 [173] Kungsholmen project	Longitudinal study	HR = 0.90; 95% CI 0.50–1.70	HR = 0.80; 95% CI 0.4–1.5	–	AF is not associated with dementia or with AD
	Follow-up for 6 years				
Ott *et al*. 1997 [171] Rotterdam study	Cross-sectional population-based	<75 years OR = 2.6; 95% CI 0.60–11.40	OR = 1.8; 95% CI 0.9–3.5	OR = 1.5; 95% CI 0.4–4.9	AF is associated more strongly with AD than with vascular dementia
		>75 years OR = 2.2; 95% CI 1.30–3.80			
Peters *et al*. 2009 [240] Hypertension in the Very Elderly	Double-blinded randomized controlled trial	HR = 1.031; 95% CI 0.619–1.718	–	–	AF is not associated with dementia
Rastas *et al*. 2007 [241] Vantaa Study	Longitudinal study	HR not available	–	–	AF is not associated with dementia
	Follow-up for 3.5 years				

OR, odds ratio; HR, hazard ratio; CI, confidence interval; AD, Alzheimer's disease; AF, atrial fibrillation.

A recently published meta-analysis of 21 studies confirmed that AF is associated with an increased risk of cognitive impairment and dementia [175]. However, the estimated relative risk of dementia was high (2.7) only amongst AF patients who experienced a stroke. In a wider population, AF was shown to increase slightly the risk of cognitive impairment (relative risk of 1.4).

Cerebral emboli have been reported in at least one-third of HF patients [176], and the risk of embolism increases in the presence of AF. Progressive asymptomatic embolism may persist for many years and can cause cerebral damage. This condition is a possible contributor to cognitive impairment [177] and has been found to be associated with AD [178]. Another cause of cognitive decline may be irregular and rapid ventricular rates which could lead to fluctuating and reduced CBF due to low cardiac output [179]. This may be especially common in the setting of acute HF secondary to rapid AF, where a combination of pulmonary oedema, hypoxia, renal failure and hospitalization may contribute to confusion in susceptible patients.

Treatment with a vitamin K antagonist or novel oral anticoagulants is recommended in patients with AF, except in rare cases in which there are no risk factors for stroke [180]. Despite national and international recommendations, almost half of patients with AD and AF do not receive anticoagulation, which may cause microemboli and further worsen cognition. The most common reason for undertreatment of AF with anticoagulants is old age [181,182]. However, there is evidence that patients with AD have an increased risk of intracranial bleeding [66], which is a serious adverse effect of vitamin K antagonists. Therefore, new ways to treat AF in patients with AD are of considerable interest [183].

## Heart valve disease

It has been suggested that aortic and mitral valve damage may contribute to AD by causing hypoperfusion of the brain [184]. In autopsy studies, valve damage was a common finding amongst patients with AD [185]. In an echocardiographic study, patients with AD were more likely to have aortic valve thickening and regurgitation compared with control subjects, patients with vascular dementia and nondemented individuals that experienced stroke [186]. Another echocardiographic examination of patients with AD showed impaired transmitral flow efficiency of diastolic filling compared with control subjects. The authors of this initial study [187], which was conducted in age-matched groups of patients with and without AD, found that the value of vortex formation time (an emerging quantitative index of cardiac health) is out of the optimal range in patients with AD. Vortex formation time is an emerging quantitative index of cardiac health. If this parameter is within optimal range it suggests efficient intraventricular blood transport.

## Sleep-disordered breathing

Sleep-disordered breathing (SDB) is common in the elderly and presents as obstructive, central or mixed sleep apnoeas characterized by pauses in breathing or shallow breaths during sleep [188,189]. Obstructive sleep apnoea is the most common type of this condition and is associated with cardiovascular morbidity and mortality [190].

The prevalence of SDB is higher in HF patients compared with the general population and affects HF patients with preserved and reduced ejection fraction to a similar extent [191,192]. It is estimated that 18% of HF patients have obstructive sleep apnoea and <1% suffer from the central form [193], although these two types of SDB may coexist in HF [194]. It seems likely that sleep apnoea syndrome leads to several pathophysiological conditions, such as tachycardia, hypertension and arrhythmia, that contribute to the development or exacerbation of HF [195]. In the presence of HF, the sensitivity of central chemoreceptors is enhanced which results in unstable ventilator control systems leading to the development of sleep apnoea [196,197]. Therefore, the relationship between HF and SDB is complex, with both conditions influencing each other.

Increasing evidence shows that SDB is also associated with cognitive decline and abnormalities of the hippocampus and synaptic plasticity, and occurs more frequently in patients with AD than in nondemented individuals [198,199]. It is debated whether SDB is involved in the aetiology of AD; it is possible that it could contribute to the development of AD through cerebral hypoxaemia, inflammation and/or oxidative stress [200–202]. Treatment with continuous positive airway pressure has been shown to slow cognitive deterioration in patients with mild and moderate AD [203] and seems to be well tolerated [204].

Alhough not yet supported by research, it seems likely that SDB may be a common comorbidity in patients with HF and AD and have a role in the pathogenesis of both conditions.

## Effects of medication

In HF with reduced ejection fraction, neurohormonal antagonists reduce symptoms, morbidity and mortality [17]. These drugs include renin–angiotensinogen–aldosterone system (RAAS) antagonists, beta-blockers and mineralocorticoid receptor antagonists [205]. Diuretics are often required in HF to reduce congestion and symptoms, but do not improve outcomes and may contribute to dehydration, hypotension and neurohormonal activation [17]. Overuse of diuretics in the elderly can lead to or worsen cognitive problems due to electrolyte disturbances, especially hyponatraemia [182]. Blocking the RAAS improves cognition in a manner that does not depend on blood pressure [206,207]. Angiotensin II receptor antagonists may have a neuroprotective effect on focal cerebral ischaemia [208]. Treatment with these agents may prevent cognitive decline by reducing the likelihood of vasoconstriction and thrombogenesis and, at the same time, potentiating vasodilatation and endothelial modulation [209]. However, although HF with preserved ejection fraction also involves neurohormonal activation, randomized controlled trials have not been able to demonstrate improved outcomes with neurohormonal antagonists. This may be due to underpowering of these studies or the fact that multiple age-related comorbidities may be relatively more dominant in HF with preserved ejection fraction than in HF with reduced ejection fraction. In a large registry-based study, RAAS antagonists do appear to be associated with improved outcomes [210].

In AD, cholinesterase inhibitors, which are the most commonly prescribed antidementia drugs[211], may have cardioprotective functions [212]. It has been reported that donepezil decreases cardiovascular mortality [213]. In patients with subclinical HF, a decrease in the level of brain natriuretic peptide has been observed as a consequence of donepezil treatment. This suggests that cholinesterase inhibitors could have a beneficial therapeutic effect in HF [214]. Similar results have been found in an observational study in which the use of cholinesterase inhibitors was associated with a 35% reduced risk of myocardial infarction in patients with AD [215].

This relationship may be explained by the fact that cholinesterase inhibitors have anti-inflammatory properties which positively impact the inflammation underlying atherosclerosis [216]. It has also been suggested that these agents have a negative chronotropic effect on the heart and increase vagal tone by augmenting acetylcholine [217,218]. This is in line with an experiment performed on animal models, where vagal nerve stimulation improved the survival after HF [219]. Furthermore, this finding is supported by beneficial effects on reducing heart rate observed with beta-blockers and ivabradine, which reduce heart rate by direct action on the sinus node and improve outcomes in HF [220] and coronary disease [221]. These findings are in contrast with current clinical practice as donepezil is not always prescribed for patients with both AD and cardiovascular disease [214]. On the other hand, excessive cholinergic stimulation, which may be caused by cholinesterase inhibitors, has been reported to cause arrhythmias, such as bradycardia, sick sinus syndrome and torsades de pointes [222].

## Discussion

Interest in ‘cardiogenic dementia’ has been renewed by evidence of cognitive decline in patients with HF. In this review, we have attempted to explain how HF contributes to the development of AD. However, not all studies included here examined patients who were diagnosed with AD, thus much evidence relates to dementia in general. The results of several studies are limited by the fact that AD often overlaps with other dementia types, such as vascular dementia. Epidemiological studies have shown that both AD and vascular dementia share similar risk factors [223], and vascular pathology has been observed in autopsy investigations in patients with AD more frequently than expected [224]. There has been a lack of consensus on integrating vascular changes into diagnostic criteria of dementia [224]; therefore, the diagnosis of AD may vary in different studies.

Reduced CBF is a well-established factor associated with cognitive impairment in HF patients, and microscopical findings support this relationship. However, the progressive pathophysiology of HF involves maladaptive neurohormonal activation, systemic inflammation, oxidative stress and impaired endothelial function [94]. It is likely that the relationship between HF and dementia is much more complex than initially thought and may reflect neurohormonal activation and systemic remodelling, rather than perfusion alone. Both HF and AD are strongly associated with advanced age. Elderly individuals usually suffer from many comorbidities that have a dual effect on AD: both affecting it directly and causing cardiovascular diseases. It has been suggested that underlying and concomitant conditions may be more important in the development of AD than reduced CBF [225]. However, the direct role of hypoperfusion versus the indirect role of neurohormonal activation or comorbidities remains unclear, and specific causes are difficult to isolate.

**Figure 3:**
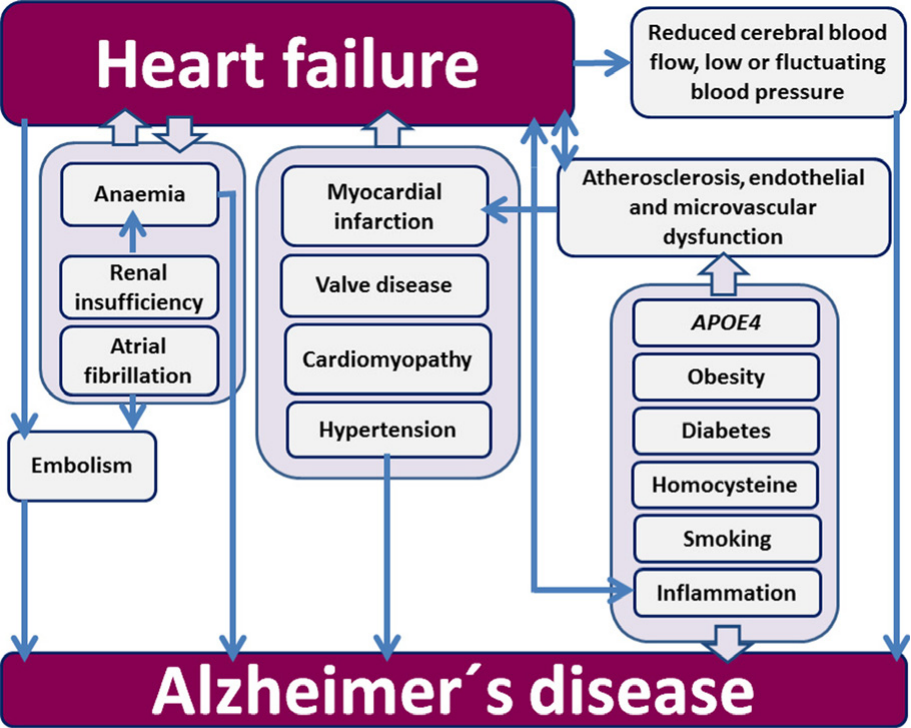
Schematic diagram of the complex relationship between heart failure and Alzheimer′s disease.

Atherosclerosis is strongly involved in the pathogenesis of HF and has been suggested to contribute to AD. Its risk factors such as hypertension, hypercholesterolaemia, *ApoE4* polymorphisms, obesity, smoking, homocysteine and chronic inflammation have been demonstrated to increase the risk of AD and may play a role in microvascular dysfunction in HF even in the absence of macrovascular coronary disease. Furthermore, AF and anaemia are involved in the pathogenesis of HF and AD in several ways: they may both precipitate and be caused by HF, as well as contribute to the onset of AD [226–228]. HF may lead to renal insufficiency and vice versa [229] and additionally kidney insufficiency contributes substantially to the development of anaemia [230]. Fig.3 shows the various ways in which these conditions are involved in the emergence of AD in HF patients.

Evidence suggests that treatment of HF may improve cognition and delay the onset of dementia. However, the therapeutic management of HF is complicated by side effects and their interaction with ageing, frailty and perhaps dementia itself. Furthermore, there is no treatment proven to retard the development of or improve outcomes in HF with preserved ejection fraction, which is the type of HF that is particularly common in the elderly and those with comorbidities such as AF.

As the relationship between HF and AD becomes increasingly important with the ageing worldwide population, more attention should be focused on the field of neurocardiology, a subspecialty concerned with associations between the heart and brain [231,232]. Awareness of cognitive impairment should challenge clinicians in primary care units as well as cardiologists, and cognition should be evaluated in patients with HF. An interdisciplinary approach towards elderly patients is recommended [233–235]. The future of neurocardiology may depend on integrating medical knowledge of interactions between chronic degenerative and cardiovascular diseases and applying this knowledge in clinical practice [236]. Better understanding of such relationships may result in benefit for elderly patients from appropriate evidence-based treatment.

## References

[1] Prince M, Bryce R, Albanese E, Wimo A, Ribeiro W, Ferri CP (2013). The global prevalence of dementia: a systematic review and metaanalysis. Alzheimers Dement.

[2] Wimo A, Jonsson L, Bond J, Prince M, Winblad B, Alzheimer Disease International (2013). The worldwide economic impact of dementia 2010. Alzheimers Dement.

[3] Wallin K, Bostrom G, Kivipelto M, Gustafson Y (2013). Risk factors for incident dementia in the very old. Int Psychogeriatr.

[4] Kivipelto M, Helkala EL, Laakso MP (2001). Midlife vascular risk factors and Alzheimer's disease in later life: longitudinal, population based study. BMJ.

[5] Kivipelto M, Ngandu T, Fratiglioni L (2005). Obesity and vascular risk factors at midlife and the risk of dementia and Alzheimer disease. Arch Neurol.

[6] Huang W, Qiu C, von Strauss E, Winblad B, Fratiglioni L (2004). APOE genotype, family history of dementia, and Alzheimer disease risk: a 6-year follow-up study. Arch Neurol.

[7] Qiu C, Winblad B, Marengoni A, Klarin I, Fastbom J, Fratiglioni L (2006). Heart failure and risk of dementia and Alzheimer disease: a population-based cohort study. Arch Intern Med.

[8] Roger VL, Go AS, Lloyd-Jones DM (2012). Heart disease and stroke statistics–2012 update: a report from the American Heart Association. Circulation.

[9] Stewart S, MacIntyre K, Hole DJ, Capewell S, McMurray JJ (2001). More ‘malignant’ than cancer? Five-year survival following a first admission for heart failure. Eur J Heart Fail.

[10] Nieminen MS, Bohm M, Cowie MR (2005). Executive summary of the guidelines on the diagnosis and treatment of acute heart failure: the Task Force on Acute Heart Failure of the European Society of Cardiology. Eur Heart J.

[11] Jessup M, Brozena S (2003). Heart failure. N Engl J Med.

[12] Chhatre S, Weiner MG, Jayadevappa R, Johnson JC (2009). Incremental burden of congestive heart failure among elderly with Alzheimer's. Aging Ment Health.

[13] Vogels RL, Scheltens P, Schroeder-Tanka JM, Weinstein HC (2007). Cognitive impairment in heart failure: a systematic review of the literature. Eur J Heart Fail.

[14] Pressler SJ, Kim J, Riley P, Ronis DL, Gradus-Pizlo I (2010). Memory dysfunction, psychomotor slowing, and decreased executive function predict mortality in patients with heart failure and low ejection fraction. J Cardiac Fail.

[15] Roher AE, Debbins JP, Malek-Ahmadi M (2012). Cerebral blood flow in Alzheimer's disease. Vasc Health Risk Manag.

[16] Hoth KF, Poppas A, Moser DJ, Paul RH, Cohen RA (2008). Cardiac dysfunction and cognition in older adults with heart failure. Cogn Behav Neurol.

[17] McMurray JJ, Adamopoulos S, Anker SD (2012). ESC Guidelines for the diagnosis and treatment of acute and chronic heart failure 2012: The Task Force for the Diagnosis and Treatment of Acute and Chronic Heart Failure 2012 of the European Society of Cardiology. Developed in collaboration with the Heart Failure Association (HFA) of the ESC. Eur Heart J.

[18] de la Torre JC (2012). Cardiovascular risk factors promote brain hypoperfusion leading to cognitive decline and dementia. Cardiovasc Psychiatry and Neurol.

[19] Forette F, Seux ML, Staessen JA (1998). Prevention of dementia in randomised double-blind placebo-controlled Systolic Hypertension in Europe (Syst-Eur) trial. Lancet.

[20] Khachaturian AS, Zandi PP, Lyketsos CG (2006). Antihypertensive medication use and incident Alzheimer disease: the Cache County Study. Arch Neurol.

[21] Brookmeyer R, Johnson E, Ziegler-Graham K, Arrighi HM (2007). Forecasting the global burden of Alzheimer's disease. Alzheimers Dement.

[22] Rocca WA, Petersen RC, Knopman DS (2011). Trends in the incidence and prevalence of Alzheimer's disease, dementia, and cognitive impairment in the United States. Alzheimers Dement.

[23] Schrijvers EM, Verhaaren BF, Koudstaal PJ, Hofman A, Ikram MA, Breteler MM (2012). Is dementia incidence declining?: trends in dementia incidence since 1990 in the Rotterdam Study. Neurology.

[24] Qiu C, von Strauss E, Backman L, Winblad B, Fratiglioni L (2013). Twenty-year changes in dementia occurrence suggest decreasing incidence in central Stockholm, Sweden. Neurology.

[25] Christensen K, Thinggaard M, Oksuzyan A (2013). Physical and cognitive functioning of people older than 90 years: a comparison of two Danish cohorts born 10 years apart. Lancet.

[26] Matthews FE, Arthur A, Barnes LE (2013). A two-decade comparison of prevalence of dementia in individuals aged 65 years and older from three geographical areas of England: results of the Cognitive Function and Ageing Study I and II. Lancet.

[27] Gustafson Y (2004). Postoperative delirium–a challenge for the orthopedic team. Acta Orthop Scand.

[28] Eriksson I, Gustafson Y, Fagerstrom L, Olofsson B (2011). Urinary tract infection in very old women is associated with delirium. Int Psychogeriatr.

[29] Laurila JV, Laakkonen ML, Tilvis RS, Pitkala KH (2008). Predisposing and precipitating factors for delirium in a frail geriatric population. J Psychosom Res.

[30] Kiely DK, Marcantonio ER, Inouye SK (2009). Persistent delirium predicts greater mortality. J Am Geriatr Soc.

[31] Mathillas J, Olofsson B, Lovheim H, Gustafson Y (2013). Thirty-day prevalence of delirium among very old people: a population-based study of very old people living at home and in institutions. Arch Gerontol Geriatr.

[32] Krogseth M, Wyller TB, Engedal K, Juliebo V (2011). Delirium is an important predictor of incident dementia among elderly hip fracture patients. Dement Geriatr Cogn Disord.

[33] Owan TE, Hodge DO, Herges RM, Jacobsen SJ, Roger VL, Redfield MM (2006). Trends in prevalence and outcome of heart failure with preserved ejection fraction. N Engl J Med.

[34] Donal E, Lund LH, Linde C (2009). Rationale and design of the Karolinska-Rennes (KaRen) prospective study of dyssynchrony in heart failure with preserved ejection fraction. Eur J Heart Fail.

[35] Donal E, Thebault C, Lund LH (2012). Heart failure with a preserved ejection fraction additive value of an exercise stress echocardiography. Eur Heart J Cardiovasc Imaging.

[36] Campbell RT, Jhund PS, Castagno D, Hawkins NM, Petrie MC, McMurray JJ (2012). What have we learned about patients with heart failure and preserved ejection fraction from DIG-PEF, CHARM-preserved, and I-PRESERVE?. J Am Coll Cardiol.

[37] (1977). Cardiogenic Dementia. Lancet.

[38] Debette S, Bauters C, Leys D, Lamblin N, Pasquier F, de Groote P (2007). Prevalence and determinants of cognitive impairment in chronic heart failure patients. Congest Heart Fail.

[39] Cohen MB, Mather PJ (2007). A review of the association between congestive heart failure and cognitive impairment. Am J Geriatr Cardiol.

[40] Jefferson AL (2010). Cardiac output as a potential risk factor for abnormal brain aging. J Alzheimers Dis.

[41] Jefferson AL, Himali JJ, Au R (2011). Relation of left ventricular ejection fraction to cognitive aging (from the Framingham Heart Study). Am J Cardiol.

[42] Zuccala G, Cattel C, Manes-Gravina E, Di Niro MG, Cocchi A, Bernabei R (1997). Left ventricular dysfunction: a clue to cognitive impairment in older patients with heart failure. J Neurol Neurosurg Psychiatry.

[43] Putzke JD, Williams MA, Daniel JF, Foley BA, Kirklin JK, Boll TJ (2000). Neuropsychological functioning among heart transplant candidates: a case control study. J Clin Exp Neuropsychol.

[44] Deshields TL, McDonough EM, Mannen RK, Miller LW (1996). Psychological and cognitive status before and after heart transplantation. Gen Hosp Psychiatry.

[45] Roman DD, Kubo SH, Ormaza S, Francis GS, Bank AJ, Shumway SJ (1997). Memory improvement following cardiac transplantation. J Clin Exp Neuropsychol.

[46] Dodson JA, Truong TT, Towle VR, Kerins G, Chaudhry SI (2013). Cognitive impairment in older adults with heart failure: prevalence, documentation, and impact on outcomes. Am J Med.

[47] Uthamalingam S, Gurm GS, Daley M, Flynn J, Capodilupo R (2011). Usefulness of acute delirium as a predictor of adverse outcomes in patients >65 years of age with acute decompensated heart failure. Am J Cardiol.

[48] Hjelm C, Brostrom A, Dahl A, Johansson B, Fredrikson M, Stromberg A (2013). Factors associated with increased risk for dementia in individuals age 80 years or older with congestive heart failure. J Cardiovasc Nurs.

[49] Roman GC (2005). Vascular dementia prevention: a risk factor analysis. Cerebrovasc Dis.

[50] Vogels RL, van der Flier WM, van Harten B (2007). Brain magnetic resonance imaging abnormalities in patients with heart failure. Eur J Heart Fail.

[51] Kumar R, Woo MA, Macey PM, Fonarow GC, Hamilton MA, Harper RM (2011). Brain axonal and myelin evaluation in heart failure. J Neurol Sci.

[52] Serber SL, Kumar R, Woo MA, Macey PM, Fonarow GC, Harper RM (2008). Cognitive test performance and brain pathology. Nurs Res.

[53] Prins ND, van der Flier WM, Brashear HR (2013). Predictors of progression from mild cognitive impairment to dementia in the placebo-arm of a clinical trial population. J Alzheimers Dis.

[54] Jefferson AL, Himali JJ, Beiser AS (2010). Cardiac index is associated with brain aging: the Framingham Heart Study. Circulation.

[55] Vasudev A, Saxby BK, O'Brien JT (2012). Relationship between cognition, magnetic resonance white matter hyperintensities, and cardiovascular autonomic changes in late-life depression. Am J Geriatr Psychiatry.

[56] Farid K, Petras S, Ducasse V (2012). Brain perfusion SPECT imaging and acetazolamide challenge in vascular cognitive impairment. Nucl Med Commun.

[57] Bronge L, Wahlund LO (2007). White matter changes in dementia: does radiology matter?. Br J Radiol.

[58] Scheibel AB, Duong TH, Jacobs R (1989). Alzheimer's disease as a capillary dementia. Ann Med.

[59] Yoshikawa T, Murase K, Oku N (2003). Heterogeneity of cerebral blood flow in Alzheimer disease and vascular dementia. AJNR Am J Neuroradiol.

[60] Brown WR, Thore CR (2011). Review: cerebral microvascular pathology in ageing and neurodegeneration. Neuropathol Appl Neurobiol.

[61] Johnson NA, Jahng GH, Weiner MW (2005). Pattern of cerebral hypoperfusion in Alzheimer disease and mild cognitive impairment measured with arterial spin-labeling MR imaging: initial experience. Radiology.

[62] Mazza M, Marano G, Traversi G, Bria P, Mazza S (2011). Primary cerebral blood flow deficiency and Alzheimer's disease: shadows and lights. J Alzheimers Dis.

[63] Ruitenberg A, den Heijer T, Bakker SL (2005). Cerebral hypoperfusion and clinical onset of dementia: the Rotterdam Study. Ann Neurol.

[64] Rombouts SA, Goekoop R, Stam CJ, Barkhof F, Scheltens P (2005). Delayed rather than decreased BOLD response as a marker for early Alzheimer's disease. NeuroImage.

[65] Hirao K, Ohnishi T, Hirata Y (2005). The prediction of rapid conversion to Alzheimer's disease in mild cognitive impairment using regional cerebral blood flow SPECT. NeuroImage.

[66] Bell RD, Zlokovic BV (2009). Neurovascular mechanisms and blood-brain barrier disorder in Alzheimer's disease. Acta Neuropathol.

[67] Jellinger KA (2002). Alzheimer disease and cerebrovascular pathology: an update. J Neural Transm.

[68] Choi BR, Kim JS, Yang YJ (2006). Factors associated with decreased cerebral blood flow in congestive heart failure secondary to idiopathic dilated cardiomyopathy. Am J Cardiol.

[69] Paulson OB, Jarden JO, Godtfredsen J, Vorstrup S (1984). Cerebral blood flow in patients with congestive heart failure treated with captopril. Am J Med.

[70] Gruhn N, Larsen FS, Boesgaard S (2001). Cerebral blood flow in patients with chronic heart failure before and after heart transplantation. Stroke.

[71] Massaro AR, Dutra AP, Almeida DR, Diniz RV, Malheiros SM (2006). Transcranial Doppler assessment of cerebral blood flow: effect of cardiac transplantation. Neurology.

[72] de la Torre JC (2008). Pathophysiology of neuronal energy crisis in Alzheimer's disease. Neuro-Degener Dis.

[73] Pirchl M, Humpel C (2009). [Does acidosis in brain play a role in Alzheimer's disease?]. Neuropsychiatr.

[74] Ishizaki T, Erickson A, Kuric E (2010). The asparaginyl endopeptidase legumain after experimental stroke. J Cereb Blood Flow Metab.

[75] Liu F, Grundke-Iqbal I, Iqbal K, Gong CX (2005). Contributions of protein phosphatases PP1, PP2A, PP2B and PP5 to the regulation of tau phosphorylation. Eur J Neurosci.

[76] Kolarova M, Garcia-Sierra F, Bartos A, Ricny J, Ripova D (2012). Structure and pathology of tau protein in Alzheimer disease. Int J Alzheimers Dis.

[77] Iqbal K, Gong CX, Liu F (2013). Hyperphosphorylation-induced tau oligomers. Front Neurol.

[78] Velliquette RA, O'Connor T, Vassar R (2005). Energy inhibition elevates beta-secretase levels and activity and is potentially amyloidogenic in APP transgenic mice: possible early events in Alzheimer's disease pathogenesis. J Neurosci.

[79] Bailey TL, Rivara CB, Rocher AB, Hof PR (2004). The nature and effects of cortical microvascular pathology in aging and Alzheimer's disease. Neurol Res.

[80] Iadecola C (2004). Neurovascular regulation in the normal brain and in Alzheimer's disease. Nat Rev Neurosci.

[81] Zlokovic BV (2008). The blood-brain barrier in health and chronic neurodegenerative disorders. Neuron.

[82] Glass CK, Saijo K, Winner B, Marchetto MC, Gage FH (2010). Mechanisms underlying inflammation in neurodegeneration. Cell.

[83] Meraz-Rios MA, Toral-Rios D, Franco-Bocanegra D, Villeda-Hernandez J, Campos-Pena V (2013). Inflammatory process in Alzheimer's Disease. Front Integr Neurosci.

[84] Farfara D, Lifshitz V, Frenkel D (2008). Neuroprotective and neurotoxic properties of glial cells in the pathogenesis of Alzheimer's disease. J Cell Mol Med.

[85] Harry GJ (2013). Microglia during development and aging. Pharmacol Ther.

[86] Bell RD, Winkler EA, Singh I (2012). Apolipoprotein E controls cerebrovascular integrity via cyclophilin A. Nature.

[87] Marchesi C, Paradis P, Schiffrin EL (2008). Role of the renin-angiotensin system in vascular inflammation. Trends Pharmacol Sci.

[88] Armulik A, Genove G, Mae M (2010). Pericytes regulate the blood-brain barrier. Nature.

[89] Ritz K, van Buchem MA, Daemen MJ (2013). The heart-brain connection: mechanistic insights and models. Neth Heart J.

[90] Arai K, Lo EH (2010). Astrocytes protect oligodendrocyte precursor cells via MEK/ERK and PI3K/Akt signaling. J Neurosci Res.

[91] Thal DR, Griffin WS, de Vos RA, Ghebremedhin E (2008). Cerebral amyloid angiopathy and its relationship to Alzheimer's disease. Acta Neuropathol.

[92] Greenberg SM, Gurol ME, Rosand J, Smith EE (2004). Amyloid angiopathy-related vascular cognitive impairment. Stroke.

[93] Okamoto Y, Yamamoto T, Kalaria RN (2012). Cerebral hypoperfusion accelerates cerebral amyloid angiopathy and promotes cortical microinfarcts. Acta Neuropathol.

[94] White M, Ducharme A, Ibrahim R (2006). Increased systemic inflammation and oxidative stress in patients with worsening congestive heart failure: improvement after short-term inotropic support. Clin Sci.

[95] Religa P, Cao R, Religa D (2013). VEGF significantly restores impaired memory behavior in Alzheimer's mice by improvement of vascular survival. Sci Rep.

[96] Grudzinska MK, Kurzejamska E, Bojakowski K (2013). Monocyte chemoattractant protein 1-mediated migration of mesenchymal stem cells is a source of intimal hyperplasia. Arterioscler Thromb Vasc Biol.

[97] Bink DI, Ritz K, Aronica E, van der Weerd L, Daemen MJ (2013). Mouse models to study the effect of cardiovascular risk factors on brain structure and cognition. J Cereb Blood Flow Metab.

[98] Shibata M, Ohtani R, Ihara M, Tomimoto H (2004). White matter lesions and glial activation in a novel mouse model of chronic cerebral hypoperfusion. Stroke.

[99] Fujita Y, Ihara M, Ushiki T (2010). Early protective effect of bone marrow mononuclear cells against ischemic white matter damage through augmentation of cerebral blood flow. Stroke.

[100] Nishio K, Ihara M, Yamasaki N (2010). A mouse model characterizing features of vascular dementia with hippocampal atrophy. Stroke.

[101] Coltman R, Spain A, Tsenkina Y (2011). Selective white matter pathology induces a specific impairment in spatial working memory. Neurobiol Aging.

[102] Mrzilkova J, Zach P, Bartos A, Tintera J, Ripova D (2012). Volumetric analysis of the pons, cerebellum and hippocampi in patients with Alzheimer's disease. Dement Geriatr Cogn Disord.

[103] Zakzanis KK, Graham SJ, Campbell Z (2003). A meta-analysis of structural and functional brain imaging in dementia of the Alzheimer's type: a neuroimaging profile. Neuropsychol Rev.

[104] Nakamura A, Rokosh DG, Paccanaro M (2001). LV systolic performance improves with development of hypertrophy after transverse aortic constriction in mice. Am J Physiol Heart Circ Physiol.

[105] Gentile MT, Poulet R, Di Pardo A (2009). Beta-amyloid deposition in brain is enhanced in mouse models of arterial hypertension. Neurobiol Aging.

[106] Kitaguchi H, Tomimoto H, Ihara M (2009). Chronic cerebral hypoperfusion accelerates amyloid beta deposition in APPSwInd transgenic mice. Brain Res.

[107] Pluta R (2000). The role of apolipoprotein E in the deposition of beta-amyloid peptide during ischemia-reperfusion brain injury. A model of early Alzheimer's disease. Ann N Y Acad Sci.

[108] Alosco ML, Brickman AM, Spitznagel MB (2012). The independent association of hypertension with cognitive function among older adults with heart failure. J Neurol Sci.

[109] Trojano L, Antonelli Incalzi R, Acanfora D (2003). Cognitive impairment: a key feature of congestive heart failure in the elderly. J Neurol.

[110] Goyal D, Macfadyen RJ, Watson RD, Lip GY (2005). Ambulatory blood pressure monitoring in heart failure: a systematic review. Eur J Heart Fail.

[111] Mosterd A, Cost B, Hoes AW (2001). The prognosis of heart failure in the general population: the Rotterdam Study. Eur Heart J.

[112] Zuccala G, Onder G, Pedone C (2001). Hypotension and cognitive impairment: selective association in patients with heart failure. Neurology.

[113] den Abeelen AS, Lagro J, van Beek A, Claassen J (2014). Impaired cerebral autoregulation and vasomotor reactivity in sporadic Alzheimer's in disease. Curr Alzheimer Res.

[114] Skoog I, Lernfelt B, Landahl S (1996). 15-year longitudinal study of blood pressure and dementia. Lancet.

[115] Qiu C, von Strauss E, Winblad B, Fratiglioni L (2004). Decline in blood pressure over time and risk of dementia: a longitudinal study from the Kungsholmen project. Stroke.

[116] Fitzgerald AA, Powers JD, Ho PM (2011). Impact of medication nonadherence on hospitalizations and mortality in heart failure. J Cardiac Fail.

[117] Sabayan B, Wijsman LW, Foster-Dingley JC (2013). Association of visit-to-visit variability in blood pressure with cognitive function in old age: prospective cohort study. BMJ.

[118] Ruland S, Aiyagari V (2007). Cerebral autoregulation and blood pressure lowering. Hypertension.

[119] Domanski MJ, Mitchell GF, Norman JE, Exner DV, Pitt B, Pfeffer MA (1999). Independent prognostic information provided by sphygmomanometrically determined pulse pressure and mean arterial pressure in patients with left ventricular dysfunction. J Am Coll Cardiol.

[120] Eklind-Cervenka M, Benson L, Dahlstrom U, Edner M, Rosenqvist M, Lund LH (2011). Association of candesartan vs losartan with all-cause mortality in patients with heart failure. JAMA.

[121] Kannel WB, Castelli WP, McNamara PM, McKee PA, Feinleib M (1972). Role of blood pressure in the development of congestive heart failure. The Framingham study. N Engl J Med.

[122] Launer LJ, Masaki K, Petrovitch H, Foley D, Havlik RJ (1995). The association between midlife blood pressure levels and late-life cognitive function. The Honolulu-Asia Aging Study. JAMA.

[123] Igase M, Kohara K, Miki T (2012). The Association between Hypertension and Dementia in the Elderly. Int J Hypertens.

[124] Applegate WB, Pressel S, Wittes J (1994). Impact of the treatment of isolated systolic hypertension on behavioral variables. Results from the systolic hypertension in the elderly program. Arch Intern Med.

[125] Lithell H, Hansson L, Skoog I (2003). The Study on Cognition and Prognosis in the Elderly (SCOPE): principal results of a randomized double-blind intervention trial. J Hypertens.

[126] Waldstein SR, Manuck SB, Ryan CM, Muldoon MF (1991). Neuropsychological correlates of hypertension: review and methodologic considerations. Psychol Bull.

[127] Paulus WJ, Tschope C (2013). A novel paradigm for heart failure with preserved ejection fraction: comorbidities drive myocardial dysfunction and remodeling through coronary microvascular endothelial inflammation. J Am Coll Cardiol.

[128] van Oijen M, de Jong FJ, Witteman JC, Hofman A, Koudstaal PJ, Breteler MM (2007). Atherosclerosis and risk for dementia. Ann Neurol.

[129] Roher AE, Tyas SL, Maarouf CL (2011). Intracranial atherosclerosis as a contributing factor to Alzheimer's disease dementia. Alzheimers Dement.

[130] Kalback W, Esh C, Castano EM (2004). Atherosclerosis, vascular amyloidosis and brain hypoperfusion in the pathogenesis of sporadic Alzheimer's disease. Neurol Res.

[131] Krishnaswamy G (2010). The inflammation paradigm and coronary artery disease: what Celsus, Virchow and gene knock outs have taught us. Cardiovascu Hematol Disord Drug Targets.

[132] Kovacic JC, Castellano JM, Fuster V (2012). The links between complex coronary disease, cerebrovascular disease, and degenerative brain disease. Ann N Y Acad Sci.

[133] Olazaran J, Ramos A, Boyano I (2014). Pattern of and risk factors for brain microbleeds in neurodegenerative dementia. Am J Alzheimers Dis Other Demen.

[134] Webber BJ, Seguin PG, Burnett DG, Clark LL, Otto JL (2012). Prevalence of and risk factors for autopsy-determined atherosclerosis among US service members, 2001–2011. JAMA.

[135] Farrer LA, Cupples LA, Haines JL (1997). Effects of age, sex, and ethnicity on the association between apolipoprotein E genotype and Alzheimer disease. A meta-analysis. APOE and Alzheimer Disease Meta Analysis Consortium. JAMA.

[136] Seshadri S, Beiser A, Selhub J (2002). Plasma homocysteine as a risk factor for dementia and Alzheimer's disease. N Engl J Med.

[137] Prince M, Cullen M, Mann A (1994). Risk factors for Alzheimer's disease and dementia: a case-control study based on the MRC elderly hypertension trial. Neurology.

[138] Gustafson D, Rothenberg E, Blennow K, Steen B, Skoog I (2003). An 18-year follow-up of overweight and risk of Alzheimer disease. Arch Intern Med.

[139] Schmidt R, Schmidt H, Curb JD, Masaki K, White LR, Launer LJ (2002). Early inflammation and dementia: a 25-year follow-up of the Honolulu-Asia Aging Study. Ann Neurol.

[140] Ott A, Stolk RP, van Harskamp F, Pols HA, Hofman A, Breteler MM (1999). Diabetes mellitus and the risk of dementia: The Rotterdam Study. Neurology.

[141] Newman AB, Fitzpatrick AL, Lopez O (2005). Dementia and Alzheimer's disease incidence in relationship to cardiovascular disease in the Cardiovascular Health Study cohort. J Am Geriatr Soc.

[142] Hofman A, Ott A, Breteler MM (1997). Atherosclerosis, apolipoprotein E, and prevalence of dementia and Alzheimer's disease in the Rotterdam Study. Lancet.

[143] Yarchoan M, Xie SX, Kling MA (2012). Cerebrovascular atherosclerosis correlates with Alzheimer pathology in neurodegenerative dementias. Brain.

[144] Beach TG, Wilson JR, Sue LI (2007). Circle of Willis atherosclerosis: association with Alzheimer's disease, neuritic plaques and neurofibrillary tangles. Acta Neuropathol.

[145] Roher AE, Esh C, Kokjohn TA (2003). Circle of willis atherosclerosis is a risk factor for sporadic Alzheimer's disease. Arterioscler Thromb Vasc Biol.

[146] Honig LS, Kukull W, Mayeux R (2005). Atherosclerosis and AD: analysis of data from the US National Alzheimer's Coordinating Center. Neurology.

[147] Kosunen O, Talasniemi S, Lehtovirta M (1995). Relation of coronary atherosclerosis and apolipoprotein E genotypes in Alzheimer patients. Stroke.

[148] Zheng L, Vinters HV, Mack WJ, Zarow C, Ellis WG, Chui HC (2013). Cerebral atherosclerosis is associated with cystic infarcts and microinfarcts but not alzheimer pathologic changes. Stroke.

[149] Dolan H, Crain B, Troncoso J, Resnick SM, Zonderman AB, Obrien RJ (2010). Atherosclerosis, dementia, and Alzheimer disease in the Baltimore Longitudinal Study of Aging cohort. Ann Neurol.

[150] Wolf PA (2012). Contributions of the Framingham Heart Study to stroke and dementia epidemiologic research at 60 years. Arch Neurol.

[151] Roberts RO, Knopman DS, Geda YE, Cha RH, Roger VL, Petersen RC (2010). Coronary heart disease is associated with non-amnestic mild cognitive impairment. Neurobiol Aging.

[152] Koschack J, Irle E (2005). Small hippocampal size in cognitively normal subjects with coronary artery disease. Neurobiol Aging.

[153] Stellos K, Katsiki N, Tatsidou P, Bigalke B, Laske C (2012). Association of platelet activation with vascular cognitive impairment: implications in dementia development?. Curr Vasc Pharmacol.

[154] Graban A, Bednarska-Makaruk M, Bochynska A, Lipczynska-Lojkowska W, Ryglewicz D, Wehr H (2009). Vascular and biochemical risk factors of vascular dementia after lacunar strokes (S-VaD) and after multiinfarcts in strategic areas (M-VaD). J Neurol Sci.

[155] Ikram MA, van Oijen M, de Jong FJ (2008). Unrecognized myocardial infarction in relation to risk of dementia and cerebral small vessel disease. Stroke.

[156] Aronson MK, Ooi WL, Morgenstern H (1990). Women, myocardial infarction, and dementia in the very old. Neurology.

[157] Petrovitch H, White L, Masaki KH (1998). Influence of myocardial infarction, coronary artery bypass surgery, and stroke on cognitive impairment in late life. Am J Cardiol.

[158] Bursi F, Rocca WA, Killian JM (2006). Heart disease and dementia: a population-based study. Am J Epidemiol.

[159] Linssen GC, Rienstra M, Jaarsma T (2011). Clinical and prognostic effects of atrial fibrillation in heart failure patients with reduced and preserved left ventricular ejection fraction. Eur J Heart Fail.

[160] Carson PE, Johnson GR, Dunkman WB, Fletcher RD, Farrell L, Cohn JN (1993). The influence of atrial fibrillation on prognosis in mild to moderate heart failure. The V-HeFT Studies. The V-HeFT VA Cooperative Studies Group. Circulation.

[161] Donal E, Lund LH, Oger E (2013). Baseline characteristics of patients with heart failure and preserved ejection fraction included in the Karolinska Rennes (KaRen) study. Arch Cardiovasc Dis.

[162] Anter E, Jessup M, Callans DJ (2009). Atrial fibrillation and heart failure: treatment considerations for a dual epidemic. Circulation.

[163] Knecht S, Oelschlager C, Duning T (2008). Atrial fibrillation in stroke-free patients is associated with memory impairment and hippocampal atrophy. Eur Heart J.

[164] Thacker EL, McKnight B, Psaty BM (2013). Atrial fibrillation and cognitive decline: a longitudinal cohort study. Neurology.

[165] Elias MF, Sullivan LM, Elias PK (2006). Atrial fibrillation is associated with lower cognitive performance in the Framingham offspring men. J Stroke Cerebrovasc Dis.

[166] Wozakowska-Kaplon B, Opolski G, Kosior D, Jaskulska-Niedziela E, Maroszynska-Dmoch E, Wlosowicz M (2009). Cognitive disorders in elderly patients with permanent atrial fibrillation. Kardiol Pol.

[167] Dublin S, Anderson ML, Haneuse SJ (2011). Atrial fibrillation and risk of dementia: a prospective cohort study. J Am Geriatr Soc.

[168] Miyasaka Y, Barnes ME, Petersen RC (2007). Risk of dementia in stroke-free patients diagnosed with atrial fibrillation: data from a community-based cohort. Eur Heart J.

[169] Cacciatore F, Testa G, Langellotto A (2012). Role of ventricular rate response on dementia in cognitively impaired elderly subjects with atrial fibrillation: a 10-year study. Dement Geriatr Cogn Disord.

[170] Forti P, Maioli F, Pisacane N, Rietti E, Montesi F, Ravaglia G (2007). Atrial fibrillation and risk of dementia in non-demented elderly subjects with and without mild cognitive impairment (MCI). Arch Gerontol Geriatr.

[171] Ott A, Breteler MM, de Bruyne MC, van Harskamp F, Grobbee DE, Hofman A (1997). Atrial fibrillation and dementia in a population-based study. The Rotterdam Study. Stroke.

[172] Bunch TJ, Weiss JP, Crandall BG (2010). Atrial fibrillation is independently associated with senile, vascular, and Alzheimer's dementia. Heart Rhythm.

[173] Marengoni A, Qiu C, Winblad B, Fratiglioni L (2011). Atrial fibrillation, stroke and dementia in the very old: a population-based study. Neurobiol Aging.

[174] Park H, Hildreth A, Thomson R, O'Connell J (2007). Non-valvular atrial fibrillation and cognitive decline: a longitudinal cohort study. Age Ageing.

[175] Kalantarian S, Stern TA, Mansour M, Ruskin JN (2013). Cognitive impairment associated with atrial fibrillation: a meta-analysis. Ann Intern Med.

[176] Lip GY, Piotrponikowski P, Andreotti F (2012). Thromboembolism and antithrombotic therapy for heart failure in sinus rhythm: an executive summary of a joint consensus document from the ESC Heart Failure Association and the ESC Working Group on Thrombosis. Thromb Haemost.

[177] Russell D (2002). Cerebral microemboli and cognitive impairment. J Neurol Sci.

[178] Purandare N, Burns A, Daly KJ (2006). Cerebral emboli as a potential cause of Alzheimer's disease and vascular dementia: case-control study. BMJ.

[179] Muqtadar H, Testai FD, Gorelick PB (2012). The dementia of cardiac disease. Curr Cardiol Rep.

[180] American College of Cardiology Foundation, American Heart Association, European Society of Cardiology (2013). Management of patients with atrial fibrillation (compilation of 2006 ACCF/AHA/ESC and 2011 ACCF/AHA/HRS recommendations): a report of the American College of Cardiology/American Heart Association Task Force on practice guidelines. Circulation.

[181] Tavassoli N, Perrin A, Berard E, Gillette S, Vellas B, Rolland Y, the RFRG (2013). Factors associated with undertreatment of atrial fibrillation in geriatric outpatients with Alzheimer disease. Am J Cardiovascu Drugs.

[182] Tulner LR, Van Campen JP, Kuper IM (2010). Reasons for undertreatment with oral anticoagulants in frail geriatric outpatients with atrial fibrillation: a prospective, descriptive study. Drugs Aging.

[183] Rabah A, Wazni O (2013). Atrial fibrillation in heart failure: catheter and surgical interventional therapies. Heart Fail Rev.

[184] de la Torre JC (2006). How do heart disease and stroke become risk factors for Alzheimer's disease?. Neurol Res.

[185] Corder EH, Ervin JF, Lockhart E, Szymanski MH, Schmechel DE, Hulette CM (2005). Cardiovascular damage in Alzheimer disease: autopsy findings from the Bryan ADRC. J Biomed Biotechnol.

[186] Reitz C, Brickman AM, Luchsinger JA, Wu WE, Small SA, Tang MX (2007). Frequency of subclinical heart disease in elderly persons with dementia. Am J Geriatr Cardiol.

[187] Belohlavek M, Jiamsripong P, Calleja AM (2009). Patients with Alzheimer disease have altered transmitral flow: echocardiographic analysis of the vortex formation time. J Ultrasound Med.

[188] Morgenthaler TI, Kagramanov V, Hanak V, Decker PA (2006). Complex sleep apnea syndrome: is it a unique clinical syndrome?. Sleep.

[189] Janssens JP, Pautex S, Hilleret H, Michel JP (2000). Sleep disordered breathing in the elderly. Aging.

[190] Shahar E, Whitney CW, Redline S (2001). Sleep-disordered breathing and cardiovascular disease: cross-sectional results of the Sleep Heart Health Study. Am J Respir Crit Care Med.

[191] Bitter T, Faber L, Hering D, Langer C, Horstkotte D, Oldenburg O (2009). Sleep-disordered breathing in heart failure with normal left ventricular ejection fraction. Eur J Heart Fail.

[192] Chan J, Sanderson J, Chan W (1997). Prevalence of sleep-disordered breathing in diastolic heart failure. Chest.

[193] Young T, Shahar E, Nieto FJ (2002). Predictors of sleep-disordered breathing in community-dwelling adults: the Sleep Heart Health Study. Arch Intern Med.

[194] Tkacova R, Niroumand M, Lorenzi-Filho G, Bradley TD (2001). Overnight shift from obstructive to central apneas in patients with heart failure: role of PCO_2_ and circulatory delay. Circulation.

[195] Mentz RJ, Fiuzat M (2014). Sleep-disordered breathing in patients with heart failure. Heart Fail Clin.

[196] Ng AC, Freedman SB (2009). Sleep disordered breathing in chronic heart failure. Heart Fail Rev.

[197] Javaheri S (1999). A mechanism of central sleep apnea in patients with heart failure. N Engl J Med.

[198] Janssens JP, Pautex S, Hilleret H, Michel JP (2000). [Respiratory sleep disorders in the elderly]. Rev Med Suisse Romande.

[199] Kinugawa K, Nguyen-Michel VH, Nguyen-Michel VH, Mariani J (2014). [Obstructive sleep apnea syndrome: a cause of cognitive disorders in the elderly?]. La Revue de Medecine Interne/Fondee par la Societe Nationale Francaise de Medecine Interne.

[200] Dyken ME, Yamada T, Glenn CL, Berger HA (2004). Obstructive sleep apnea associated with cerebral hypoxemia and death. Neurology.

[201] Daulatzai MA (2013). Death by a thousand cuts in Alzheimer's disease: hypoxia–the prodrome. Neurotox Res.

[202] Daulatzai MA (2012). Pathogenesis of cognitive dysfunction in patients with obstructive sleep apnea: a hypothesis with emphasis on the nucleus tractus solitarius. Sleep Disord.

[203] Cooke JR, Ayalon L, Palmer BW (2009). Sustained use of CPAP slows deterioration of cognition, sleep, and mood in patients with Alzheimer's disease and obstructive sleep apnea: a preliminary study. J Clin Sleep Med.

[204] Ayalon L, Ancoli-Israel S, Stepnowsky C (2006). Adherence to continuous positive airway pressure treatment in patients with Alzheimer's disease and obstructive sleep apnea. Am J Geriatr Psychiatry.

[205] Jonsson A, Edner M, Alehagen U, Dahlstrom U (2010). Heart failure registry: a valuable tool for improving the management of patients with heart failure. Eur J Heart Fail.

[206] Trialists C, Turnbull F, Neal B, Blood Pressure Lowering Treatment (2007). Blood pressure-dependent and independent effects of agents that inhibit the renin-angiotensin system. J Hypertens.

[207] Soto ME, van Kan GA, Nourhashemi F (2013). Angiotensin-converting enzyme inhibitors and Alzheimer's disease progression in older adults: results from the Reseau sur la Maladie d'Alzheimer Francais Cohort. J Am Geriatr Soc.

[208] Krikov M, Thone-Reineke C, Muller S, Villringer A, Unger T (2008). Candesartan but not ramipril pretreatment improves outcome after stroke and stimulates neurotrophin BNDF/TrkB system in rats. J Hypertens.

[209] Fournier A, Messerli FH, Achard JM, Fernandez L (2004). Cerebroprotection mediated by angiotensin II: a hypothesis supported by recent randomized clinical trials. J Am Coll Cardiol.

[210] Lund LH, Benson L, Dahlstrom U, Edner M (2012). Association between use of renin-angiotensin system antagonists and mortality in patients with heart failure and preserved ejection fraction. JAMA.

[211] Johnell K, Religa D, Eriksdotter M (2013). Differences in drug therapy between dementia disorders in the Swedish dementia registry: a nationwide study of over 7,000 patients. Dement Geriatr Cogn Disord.

[212] Handa T, Katare RG, Kakinuma Y (2009). Anti-Alzheimer's drug, donepezil, markedly improves long-term survival after chronic heart failure in mice. J Cardiac Fail.

[213] Sato K, Urbano R, Yu C (2010). The effect of donepezil treatment on cardiovascular mortality. Clin Pharmacol Ther.

[214] Kubo T, Sato T, Noguchi T (2012). Influences of donepezil on cardiovascular system–possible therapeutic benefits for heart failure–donepezil cardiac test registry (DOCTER) study. J Cardiovasc Pharmacol.

[215] Nordstrom P, Religa D, Wimo A, Winblad B, Eriksdotter M (2013). The use of cholinesterase inhibitors and the risk of myocardial infarction and death: a nationwide cohort study in subjects with Alzheimer's disease. Eur Heart J.

[216] Reale M, Iarlori C, Gambi F, Lucci I, Salvatore M, Gambi D (2005). Acetylcholinesterase inhibitors effects on oncostatin-M, interleukin-1 beta and interleukin-6 release from lymphocytes of Alzheimer's disease patients. Exp Gerontol.

[217] Park-Wyllie LY, Mamdani MM, Li P, Gill SS, Laupacis A, Juurlink DN (2009). Cholinesterase inhibitors and hospitalization for bradycardia: a population-based study. PLoS Med.

[218] Okazaki Y, Zheng C, Li M, Sugimachi M (2010). Effect of the cholinesterase inhibitor donepezil on cardiac remodeling and autonomic balance in rats with heart failure. J Physiol Sci.

[219] Li M, Zheng C, Sato T, Kawada T, Sugimachi M, Sunagawa K (2004). Vagal nerve stimulation markedly improves long-term survival after chronic heart failure in rats. Circulation.

[220] Swedberg K, Komajda M, Bohm M (2010). Ivabradine and outcomes in chronic heart failure (SHIFT): a randomised placebo-controlled study. Lancet.

[221] Fox K, Ford I, Steg PG, Tendera M, Robertson M, Ferrari R, BEAUTIFUL Investigators (2008). Heart rate as a prognostic risk factor in patients with coronary artery disease and left-ventricular systolic dysfunction (BEAUTIFUL): a subgroup analysis of a randomised controlled trial. Lancet.

[222] Tanaka A, Koga S, Hiramatsu Y (2009). Donepezil-induced adverse side effects of cardiac rhythm: 2 cases report of atrioventricular block and Torsade de Pointes. Intern Med.

[223] Tolppanen AM, Solomon A, Soininen H, Kivipelto M (2012). Midlife vascular risk factors and Alzheimer's disease: evidence from epidemiological studies. J Alzheimers Dis.

[224] Kling MA, Trojanowski JQ, Wolk DA, Lee VM, Arnold SE (2013). Vascular disease and dementias: paradigm shifts to drive research in new directions. Alzheimers Dement.

[225] Haring B, Leng X, Robinson J (2013). Cardiovascular disease and cognitive decline in postmenopausal women: results from the Women's Health Initiative Memory Study. J Am Heart Assoc.

[226] Shah RC, Buchman AS, Wilson RS, Leurgans SE, Bennett DA (2011). Hemoglobin level in older persons and incident Alzheimer disease: prospective cohort analysis. Neurology.

[227] Burashnikov A, Di Diego JM, Sicouri S (2014). A temporal window of vulnerability for development of atrial fibrillation with advancing heart failure. Eur J Heart Fail.

[228] von Haehling S, Anker MS, Jankowska EA, Ponikowski P, Anker SD (2012). Anemia in chronic heart failure: can we treat? What to treat?. Heart Fail Rev.

[229] Palazzuoli A, Antonelli G, Nuti R (2011). Anemia in Cardio-Renal Syndrome: clinical impact and pathophysiologic mechanisms. Heart Fail Rev.

[230] Santoro A (2002). Anemia in renal insufficiency. Rev Clin Exp Hematol.

[231] Sherman DG, Hart RG, Shi F (1987). Heart-brain interactions: neurocardiology or cardioneurology comes of age. Mayo Clin Proc.

[232] Natelson BH (1985). Neurocardiology. An interdisciplinary area for the 80s. Arch Neurol.

[233] Rojas-Fernandez CH, Patel T, Lee L (2014). An interdisciplinary memory clinic: a novel practice setting for pharmacists in primary care in Ontario, Canada. Ann Pharmacother.

[234] Gysan DB, Albus C, Riedel R (2012). [CorBene: a new model for collaborative care of patients with congestive heart failure]. Herz.

[235] Leventhal ME, Denhaerynck K, Brunner-La Rocca HP (2011). Swiss Interdisciplinary Management Programme for Heart Failure (SWIM-HF): a randomised controlled trial study of an outpatient inter-professional management programme for heart failure patients in Switzerland. Swiss Med Wkly.

[236] van der Wall EE, van Gilst WH (2013). Neurocardiology: close interaction between heart and brain. Neth Heart J.

[237] Luoto TM, Haikonen S, Haapasalo H (2009). Large vessel cerebral atherosclerosis is not in direct association with neuropathological lesions of Alzheimer's disease. Eur Neurol.

[238] Zheng L, Vinters HV, Mack WJ, Zarow C, Ellis WG, Chui HC (2013). Cerebral atherosclerosis is associated with cystic infarcts and microinfarcts but not Alzheimer pathologic changes. Stroke.

[239] Forti P, Maioli F, Pisacane N, Rietti E, Montesi F, Ravaglia G (2006). Atrial fibrillation and risk of dementia in non-demented elderly subjects with and without mild cognitive impairment. Neurol Res.

[240] Peters R, Poulter R, Beckett N (2009). Cardiovascular and biochemical risk factors for incident dementia in the Hypertension in the Very Elderly Trial. J Hypertens.

[241] Rastas S, Pirttila T, Mattila K (2010). Vascular risk factors and dementia in the general population aged >85 years: prospective population-based study. Neurobiol Aging.

